# Milk Leptin Surge and Biological Rhythms of Leptin and Other Regulatory Proteins in Breastmilk

**DOI:** 10.1371/journal.pone.0145376

**Published:** 2015-12-17

**Authors:** Yuriy Nozhenko, Madhu Asnani-Kishnani, Ana M. Rodríguez, Andreu Palou

**Affiliations:** Laboratory of Molecular Biology, Nutrition and Biotechnology (Nutrigenomics), University of the Balearic Islands (UIB) and CIBER Fisiopatología de la Obesidad y Nutrición (CIBEROBN), Palma de Mallorca, Balearic Islands, Spain; Kent State University, UNITED STATES

## Abstract

A significant number of chronic diseases are linked to perinatal nutrition, and prevention may be associated to naturally occurring components of breast milk. One key hormone in breast milk is leptin, related with the protection from obesity in the adulthood, thus knowing its changes through the day or lactation is crucial. We aimed to investigate the daily rhythms in the milk levels of leptin, together with other two related hormones, ghrelin and adiponectin, during lactation (days 5, 10 and 15) in rat dams, and the relation with morphometric parameters (dams and pups). Summarizing the main results, the existence of biological rhythms, but not daily and maybe circasemidian, was confirmed for the three hormones at the earliest period of lactation. The correlations performed generally showed a possible dependence of milk hormone levels on plasma levels at the early phase of lactation, while with the progression of lactation this dependence may fade and the hormone levels are suggested to be more dependent on mammary gland production/maturation. There was also a correlation between milk leptin and adiponectin levels, especially in the first half of lactation, suggesting a possible parallel regulation. Interestingly, we describe a milk leptin surge around the mid of lactation (at day 10) which may be related with pup´s growth (males and females) and with the well-known (in the literature) plasma leptin surge in pups. All this knowledge may be crucial for future applications in the development of formula milk and in relation with the role of leptin surge during lactation.

## Introduction

The association between numerous chronic diseases, including obesity, with early nutrition (both during the prenatal and postnatal periods) has been established, and is related to the term metabolic “programming” [1–3], where the role of key components such as hormones during the early period of development may be key in pre-programing brain development and body functions, thus influencing the risk for diseases in the adulthood [4]. In this sense, many studies indicate that breastfeeding protects against the development of obesity and related disorders in later life [5], and the search of the possible milk components responsible of this protective effects is a focus of interest.

Leptin is crucial in the suggested protection from obesity later on in life attributed to breastfeeding, given the recently described function of orally taken physiological amounts of leptin during lactation (which is absorbed by the pup´s stomach) in reducing the predisposition to obesity in the adulthood [6–9], thus becoming an essential nutrient in early life. A cause-effect relationship has been shown in animals [6–9], and indirect evidence has also been provided in humans [10–13]. Adipose tissue is the main source of leptin, although extra-adipose leptin is synthesized by other tissues, including the mammary gland [6, 14]. Moreover, the transference of leptin from maternal circulation to breast milk and to neonatal blood has been shown, with metabolic effects in the infant [15]. Studies in different mammals have shown that leptin gene expression is regulated during gestation and lactation [16]. For instance, in humans, serum leptin levels are increased towards the 36^th^ week of pregnancy, with postpartum normalization [17]. One important concept is the “leptin surge”, a transient peak of increased plasma leptin levels in pups during neonatal period suggested to have a role in early postnatal growth, involved in the development and function of the neuroendocrine axis [18–20]. According to this, leptin has been directly related with the neonatal development of hypothalamic neuronal connections and the above-mentioned physiological oral supplementation during lactation can even reverse the adverse metabolic effects of calorie restriction (in the hypothalamus and other tissues) during pregnancy [5, 21, 22]. Given all these facts, not only leptin’s presence in milk should be considered as important, but also its possible daily changes throughout the day in milk, since the circadian or the daily oscillations in leptin and related molecules in different tissues and circulation may be linked to the pathogenesis of obesity and related disorders acting, among others, on brain and regulating satiety and feeding behaviour [23, 24].

Other two interesting peptide hormones present in breast milk and provided to infants by mothers are ghrelin and adiponectin, which may contribute, together with leptin, to long-term control of appetite and may be also involved in the protection against obesity [5]. Ghrelin was identified as an endogenous ligand for the growth hormone secretagogue receptor type 1a (GHS-R 1a) [25] and is mainly produced by the stomach, but also by other tissues, including breast tissue [26]. Milk ghrelin levels increase during lactation and are significantly correlated with serum ghrelin concentrations in breast feeding infants [27]. Besides a stimulating growth hormone, ghrelin is an important orexigenic hormone and also regulates, among others, glucose and lipid metabolism and insulin sensitivity, playing an important role in energy homeostasis and in fat deposition and body weight gain [28, 29]. Adiponectin was described as an adipose tissue-specific secreted protein and is a serum-abundant protein [30]. It is known to have several positive health effects, as insulin sensitising actions, and anti-inflammatory and anti-apoptotic effects in different cell types [31]. Given the adiponectin presence in breast milk [32] and the expression of adiponectin receptors in the small intestine of mice since early development stages [33], it may affect infant growth and development. Along these lines, significant correlations between serum adiponectin levels in mothers and levels in milk and infants have been reported, also suggesting a possible link with neonatal development [34].

Different studies have shown circadian or daily rhythms (and related physiological changes) in breast milk nutrients or hormones, such as melatonin [35], tryptophan [36] and nucleotides [37]. As explained above, leptin is important in the early postnatal development and programming, a role also suggested for ghrelin and adiponectin. Under this panorama, we aimed to investigate the possible daily rhythms of breast milk leptin, together with ghrelin, and adiponectin, throughout lactation in rat dams, as well as the correlation between milk and plasma levels with morphometric parameters (of dams and pups). Such type of knowledge could be useful for possible future applications, such as the development of healthier and more physiologically adapted formula milk.

## Materials and Methods

### Animals and experimental design

The study was performed using 30 different dams. 3-month-old virgin female Wistar rats, weighing between 200g and 225g, were mated with male rats (Charles River Laboratories, Barcelona, Spain). The day when sperm was found in vaginal smears was defined as day 0 of pregnancy. Once fertilized, each female was placed in an individual cage with free access to water and standard laboratory chow (3 kcal/g, with 2.9% calories from fat, Panlab, Barcelona, Spain), under a lighting period of 12h light—12h darkness (lights on at 08:00 h) and a temperature of 22±1°C; the usual light intensity in the animal house during the lighting period was 400 lux (measured at 1m of distance from the roof). At day 1 after delivery, excess pups in each litter were removed in order to keep 10 pups per dam (five males and five females). Body weight and food intake of dams and offspring were followed until postnatal day 15.

### Milk and blood sample collection

Milk and blood samples of each dam were collected on days 5, 10 and 15 of lactation, and under *ad libitum* feeding conditions. At each of the indicated days, all of the dams were randomly divided in six different time groups of five rats (in order to avoid genetic background interference in the recollection time), in accordance to the specific time points at which samples were taken: 8:00, 12:00, 16:00, 20:00, 24:00, and 4:00 hours. Samples collected during the dark period were done under a dim red light. For plasma isolation, blood samples (over 400–600 μl approximately) were collected from the saphenous vein in heparinized containers and then centrifuged at 1000*g* for 10 min to obtain the plasma, which was subsequently stored at −20°C until its use. For milk collection, four hours before each extraction, dams were separated from nursing pups to guarantee that mammary glands were full of milk. Milk samples (ranging from 0.2 to 0.6 ml approx.) were collected by hand expression, as previously described [38] [39], and kept frozen at -80°C in microvials until hormone determination. The animal protocol followed in this study was reviewed and approved by the Bioethical Committee of University of Balearic Islands, and guidelines for the use and care of laboratory animals of the University were followed.

### Leptin, adiponectin and ghrelin quantification

Leptin concentration was measured with a mouse leptin enzyme-linked immunosorbent assay (ELISA) kit (R&D Systems, Minneapolis, MN, USA) (sensitivity: 22 pg/ml; intra-assay variation 3.3–4.3%); while adiponectin, octanoyl-Ser3-ghrelin (active form) and Des-Octanoyl-Ser3-ghrelin (inactive form) concentrations were measured with a rat enzyme immunosorbent assay kit (Phoenix Europe GmbH, Karlsruhe, Germany) (sensitivity: 0.12 ng/ml for both adiponectin and ghrelin kits; intra-assay variation 4.8–6.6% and 5–7% for adiponectin and ghrelin kits respectively), following their respective manufacturer´s instructions. Due to the big number of samples to analyse, the measurements were done once for each sample.

### Statistical analysis

Data are expressed as mean ± SEM. (*n* = 5 or *n = 30*, depending on analysis). One-way analysis of variance (ANOVA) was performed, and individual means were compared with least significant difference (LSD) *post hoc* test, for a first general analysis of the absence of rhythmicity (null hypothesis) or the lack of uniformity (alternative hypothesis). Thereafter, the cosinor method was used to assess the possibility of the existence of biological rhythms (educed rhythms in this case), as reviewed in [40]. In the correlation analyses, significant correlations were assessed by Pearson's correlation coefficients. The analyses were performed with SPSS for Windows (SPSS, Chicago, IL) for the ANOVA and correlation tests. For the cosinor analyses, the software used was from the Circadian Rhythm Laboratory (R. Refinetti) downloaded from http://www.circadian.org (accessed: November 2015). *p* < 0.05 was the threshold of significance.

## Results

### Biological rhythms in milk and plasma leptin levels at days 5, 10 and 15 of lactation

**Fig 1:**
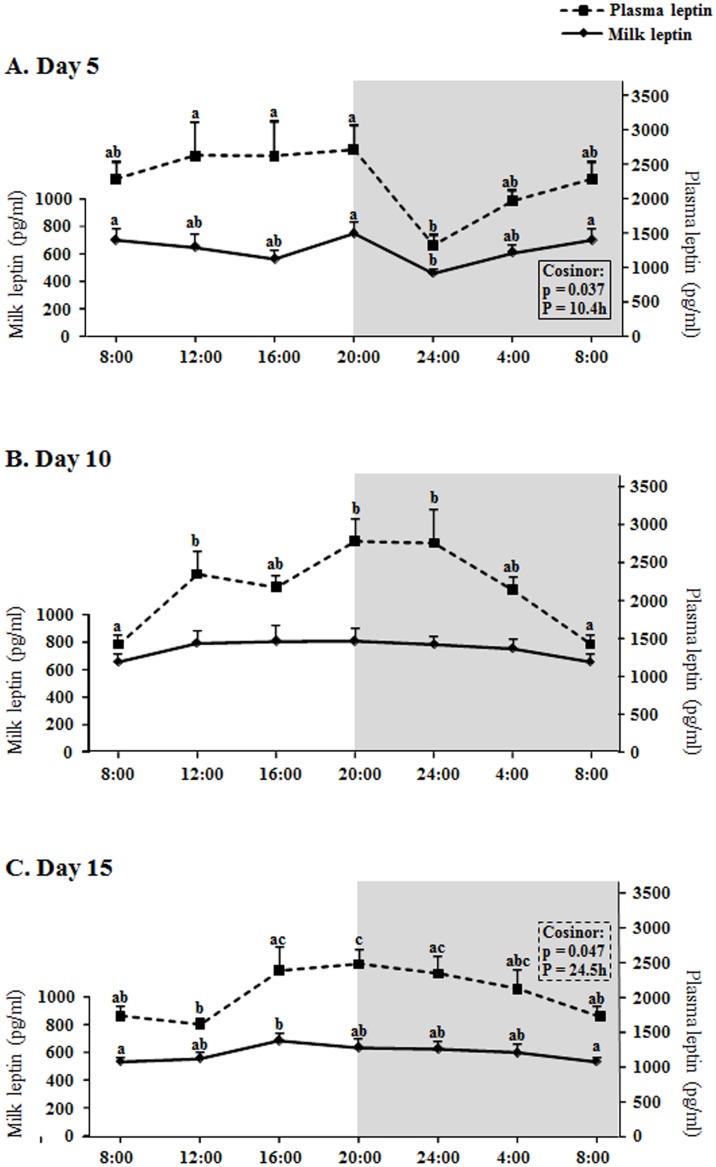
Circadian changes of milk and plasma leptin concentration. A: day 5, B: day 10, C: day 15 of lactation. X axis: time points. The shading part of the graphs indicates the dark period. The result of each time-point represents the mean value ± SEM of 5 dams. ANOVA analysis was performed, followed by LSD test for *post hoc* comparisons (a≠b≠c) for a first general analysis of the absence of rhythmicity or the lack of uniformity. Thereafter, the cosinor method was used to detect rhythmicity. *p* < 0.05 was the threshold of significance. P, period of rhythmicity obtained by the cosinor analysis.

Leptin concentration in milk and plasma in the different days and hours studied ranged (average) between 460 and 810 pg/ml and 1310 and 2780 pg/ml respectively (Fig 1), similarly to previous studies [9]; therefore, leptin levels in plasma are about 3.5-fold higher than in milk. Regarding the main question about the existence of a daily rhythm in milk leptin levels, the results show that there is rhythmicity, but not a daily rhythm, in leptin concentration in milk at the earliest period of lactation studies (day 5), with one peak (the highest levels) at the beginning of the dark period and another peak at the beginning of the light period (a bimodal pattern); in fact the cosinor analysis (significance p = 0.037) revealed that the period (P) of the rhythmicity was about half a day (P = 10.4h) (Fig 1A). Although there was not significant rhythmicity in the leptin circulating levels at day 5, the daily curve of milk leptin levels was partly parallel to the oscillations seen in plasma, showing both (milk and plasma curves) the lowest value at 24:00. At day 10, the milk leptin levels were kept constant throughout the day, with some oscillations in the plasma concentration (Fig 1B), increasing during the light phase, reaching the highest levels during the first hours of the dark phase and decreasing afterwards, but without actual rhythmicity. At day 15, the milk leptin concentration was also kept essentially constant, with a small increase at 16:00 with respect to 8:00, with a fluctuation partly parallel to the plasma levels, which showed a true daily rhythm, with the highest leptin levels from 16:00 to 24:00, especially at the beginning of the dark phase and with a period around 24h as given by the cosinor analysis (Fig 1C); moreover, the adjustment of the changes of plasma leptin at day 15 to a cosine wave was significant (p<0.01), with a mean of 2109.0 pg/ml, an amplitude of 432.0 pg/ml, and an acrophase of 21.2h.

### Biological rhythms in milk and plasma ghrelin levels at days 5, 10 and 15 of lactation

**Fig 2:**
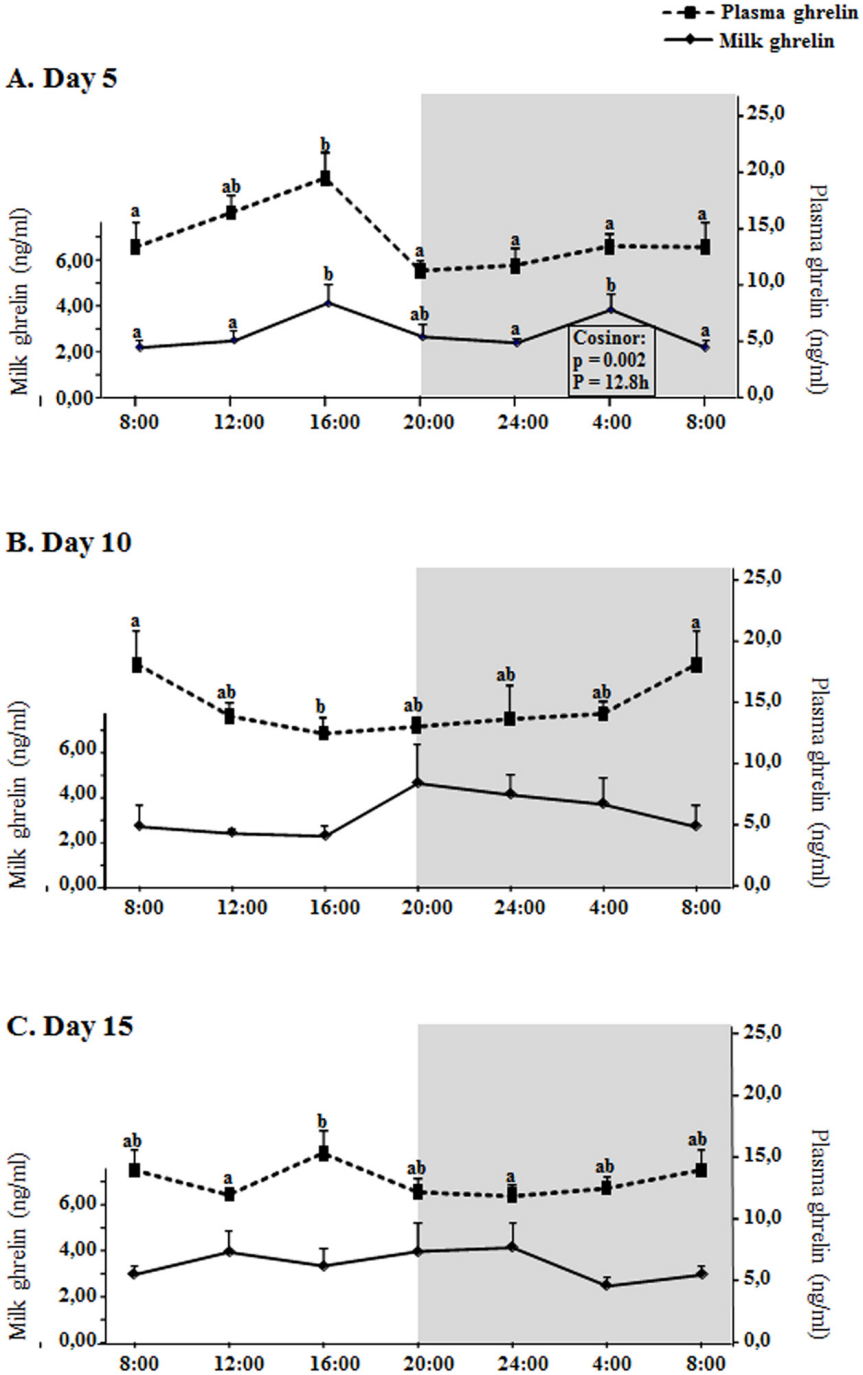
Circadian changes of milk and plasma ghrelin concentration. A: day 5, B: day 10, C: day 15 of lactation. X axis: time points. The shading part of the graphs indicates the dark period. The result of each time-point represents the mean value ± SEM of 5 dams. ANOVA, LSD test (a≠b) and cosinor analysis were performed as explained in fig 1 and materials and methods section.

Ghrelin levels, in both milk and plasma, were higher than leptin levels, about one order of magnitude, and ranged (average) from 2,2 to 4,6 ng/ml in milk and from 11,8 to 19,5 in plasma (Fig 2), thus plasma ghrelin levels were about 5-fold milk levels. Ghrelin concentration in milk showed significant rhythmicity only at day 5, also with a bimodal pattern (as leptin) but peaking at 16:00 and 4:00 (Fig 2A), with a period of half a day (P = 12.8h). Although there was not significant rhythmicity in the plasma levels, the milk ghrelin rhythm was closely parallel to the plasma changes at day 5, especially during the light phase, with a coincident peak of increased ghrelin concentration at 16:00. No significant rhythmicity was found in milk or plasma levels of ghrelin either at day 10 or at day 15 (Fig 2B and 2C).

### Biological rhythms in milk and plasma adiponectin levels at days 5, 10 and 15 of lactation

**Fig 3:**
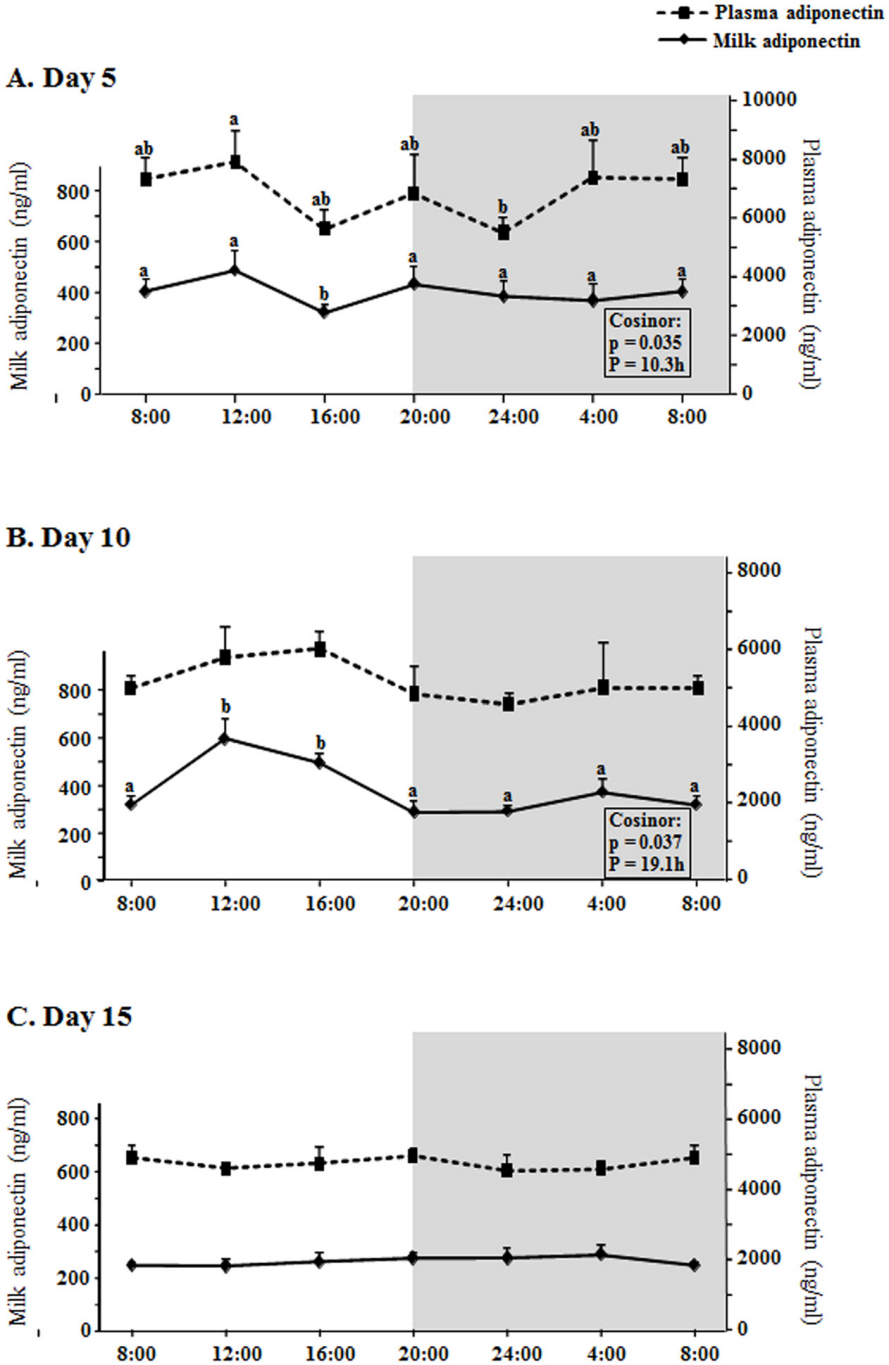
Circadian changes of milk and plasma adiponectin concentration. A: day 5, B: day 10, C: day 15 of lactation. X axis: time points. The shading part of the graphs indicates the dark period. The result of each time-point represents the mean value ± SEM of 5 dams. ANOVA, LSD test (a≠b) and cosinor analysis were performed as explained in fig 1 and materials and methods section.

**Fig 4:**
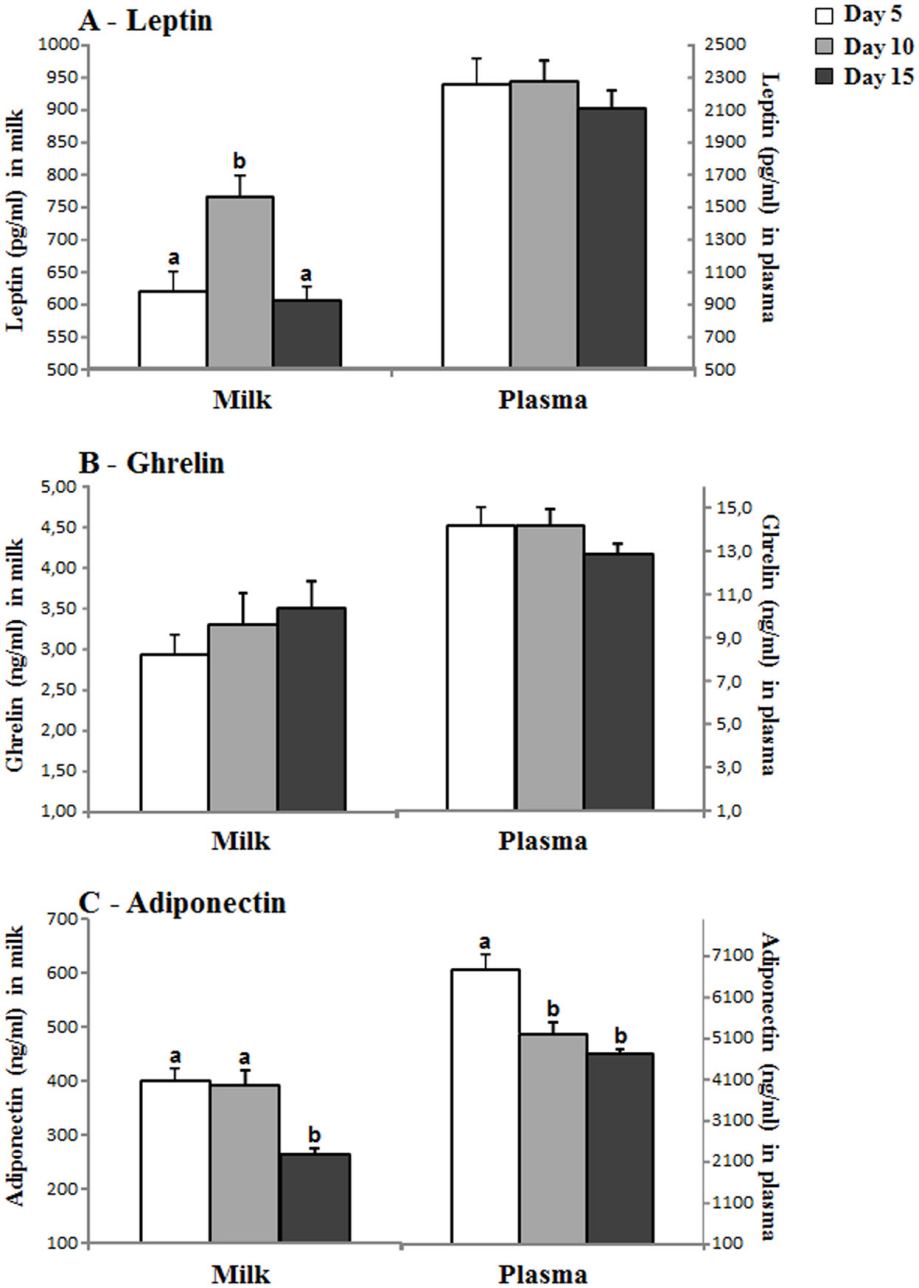
Changes in milk and plasma leptin (A), ghrelin (B) and adiponectin (C) concentration throughout lactation (days 5, 10 and 15). Each bar in the graphs represents the mean value ± SEM of 30 dams. ANOVA analysis was performed, followed by LSD test for *post hoc* comparisons (a≠b).

Previous studies have shown that breast milk adiponectin is around 20 to 100 times higher than other hormones, such as leptin and ghrelin [5]. Similar data is shown in our study, which shows that milk adiponectin ranges (average) from 240 to 590 ng/ml, and also that plasma adiponectin ranges from 4520 to 7890 ng/ml, thus also showing higher levels than leptin and ghrelin in both plasma and milk, of about two and three orders of magnitude with respect ghrelin and leptin respectively (Figs 3 and 4). With respect to the rhythms studied here, adiponectin levels showed significant rhythmicity in milk both at day 5 and 10 (Fig 3A and 3B). At day 5, the changes of milk adiponectin partially reflected the changes also observed in plasma levels, especially during the light phase (Fig 3A), although with a significant decrease at 16:00. The cosinor analysis revealed a rhythm, with a period of 10.3h (considering the measured times, two peaks at 12:00 and 20:00 are observed in the figure), i.e. again with a period of approximately half a day, like happened with leptin and adiponectin. With respect to day 10 (Fig 3B), there was an increase in milk adiponectin levels peaking at 12:00 and 16:00 and decreasing afterwards, not parallel to changes in plasma levels, which in fact did not oscillate. The cosinor analysis revealed the existence of a rhythmicity in milk adiponectin levels with a period of 19.1h, but again not a daily rhythm (in fact, the adjustment to a daily cosine wave is not significant –data not shown). At day 15, neither plasma nor milk adiponectin levels changed throughout the day.

### Changes in milk and plasma leptin, ghrelin and adiponectin mean levels comparing different days of lactation (5, 10, 15)

As described above, plasma adiponectin shows higher levels than leptin and ghrelin in both plasma and milk, of about two and three orders of magnitude with respect to ghrelin and leptin respectively (Fig 4). We also calculated the mean value of the levels of each hormone at the different days studied: day 5, 10 and 15. The results show that while ghrelin (both milk and plasma) and plasma leptin in dams did not change when comparing the different days of lactation studied, there were significant changes for both milk and plasma adiponectin and for milk leptin. The levels of milk adiponectin descended at day 15 with respect to days 5 and 10, while the plasma levels were already significantly lower at day 10 (and similar at day 15) with respect to day 5. Interestingly, the pattern of milk leptin levels changes was different, since there was a significant peak of increased levels at day 10 compared to days 5 and 15 (Fig 4A).

### Correlations between leptin, ghrelin and adiponectin milk and plasma levels and with weight and caloric intake parameters

The significant bivariate correlations between the milk and circulating levels of the hormones studied as well as with the weight of dams at days 5, 10 and 15 are given in Tables 1–3. At day 5 (Table 1), milk leptin levels were positively correlated with plasma leptin, milk and plasma adiponectin and dam´s weight; the same happened at day 10 (Table 2), except for the correlation with plasma adiponectin; and at day 15 (Table 3) the only correlation for milk leptin persisting was with plasma leptin. Plasma leptin was also correlated with milk ghrelin (day 10), milk adiponectin (days 5 and 15), plasma adiponectin (day 15) and dam’s weight (at all the days studied: 5, 10 and 15). Milk ghrelin was only correlated with plasma ghrelin at day 5 and no more correlations were found for milk or plasma ghrelin. Apart from the correlations reported above, milk adiponectin was also correlated with plasma adiponectin at all the days studied (5, 10 and 15) and with dam’s weight only at day 5. Plasma adiponectin was also correlated with dam’s weight at day 5.

**Table 1:** Correlation matrix of the milk and plasma levels of leptin, ghrelin and adiponectin, and body weight of dams (30 animals) at day 5 of lactation.

Day 5	Milk Leptin	Plasma Leptin	Milk Ghrelin	Plasma Ghrelin	Milk Adiponectin	Plasma Adiponectin	Dam weight
**Milk Leptin**	1						
**Plasma Leptin**	**0.626** **	1					
**Milk Ghrelin**	0.195	0.062	1				
**Plasma Ghrelin**	0.224	0.172	**0.427** *	1			
**Milk Adiponectin**	**0.625** **	**0.423** *	-0.006	0.124	1		
**Plasma Adiponectin**	**0.579** **	0.334	0.213	0.175	**0.782** **	1	
**Dam weight**	**0.708** **	**0.683** **	0.059	0.126	**0.537** **	**0.374** *	1

Values are Pearson’s correlation indexes.

*Significant correlation at p<0.05 level,

**significant correlation at p<0.01 levels (bilateral).

**Table 2:** Correlation matrix of the milk and plasma levels of leptin, ghrelin and adiponectin, and body weight of dams (30 animals) at day 10 of lactation.

Day 10	Milk Leptin	Plasma Leptin	Milk Ghrelin	Plasma Ghrelin	Milk Adiponectin	Plasma Adiponectin	Dam weight
**Milk Leptin**	1						
**Plasma Leptin**	**0.562** **	1					
**Milk Ghrelin**	0.098	**0.398** *	1				
**Plasma Ghrelin**	0.207	0.041	-0.063	1			
**Milk Adiponectin**	**0.441** *	0.141	-0.245	0.139	1		
**Plasma Adiponectin**	0.131	0.204	-0.282	0.236	**0.548** **	1	
**Dam weight**	**0.429** *	**0.489** **	0.256	-0.012	0.138	0.082	1

Values are Pearson’s correlation indexes.

*Significant correlation at p<0.05 level,

**significant correlation at p<0.01 levels (bilateral).

**Table 3:** Correlation matrix of the milk and plasma levels of leptin, ghrelin and adiponectin, and body weight of dams (30 animals) at day 15 of lactation.

Day 15	Milk Leptin	Plasma Leptin	Milk Ghrelin	Plasma Ghrelin	Milk Adiponectin	Plasma Adiponectin	Dam weight
**Milk Leptin**	1						
**Plasma Leptin**	**0.473** **	1					
**Milk Ghrelin**	0.121	-0.124	1				
**Plasma Ghrelin**	0.280	0.269	0.018	1			
**Milk Adiponectin**	0.151	**0.387** *	-0.236	0.220	1		
**Plasma Adiponectin**	0.013	**0.474** **	-0.228	0.339	**0.603** **	1	
**Dam weight**	0.311	**0.552** **	0.091	0.372	-0.019	0.138	1

Values are Pearson’s correlation indexes.

*Significant correlation at p<0.05 level,

**significant correlation at p<0.01 levels (bilateral).

We also analysed the correlation between the total mean values (for each dam) through lactation of each of the three hormones in milk and plasma and the cumulative caloric intake of the dams and the weight increase of dams and pups (males and females) (Table 4). Milk leptin levels were positively correlated with plasma leptin and milk adiponectin levels, dam´s calorie intake and dam´s weight at day 15. Plasma leptin was also positively correlated with milk and plasma adiponectin and dam´s calorie intake and day 15-weight. Milk ghrelin was not correlated with any parameter considered in this analysis, while plasma ghrelin was only positively correlated with dam´s weight increase. Milk adiponectin was also positively correlated with plasma adiponectin and dam´s day 15-weight, and plasma adiponectin was positively correlated with dam´s’ day 15-weight as well. There was a negative correlation between dam´s calorie intake and dam´s weight increase, and a positive correlation between dam´s calorie intake and both male and female weight increase (pups), accompanied with a concomitant positive correlation between male and female pup´s weight increase.

**Table 4:** Correlation matrix of the total mean values of the milk and plasma levels of the three hormones and the cumulative caloric intake of the dams and the weight and weight increase of dams and pups (males and females).

	Milk leptin	Plasma leptin	Milk ghrelin	Plasma ghrelin	Milk adiponectin	Plasma adiponectin	Dams’ caloric intake	Dams’ weight (day 15)	Dams’ weight increase	Male pups’ weight increase	Female pups’ weight incrase
**Milk leptin**	1										
**Plasma leptin**	**0.776** **	1									
**Milk grhelin**	0.048	0.142	1								
**Plasma grhelin**	0.295	0.311	0.206	1							
**Milk adiponectin**	**0.475** **	**0.434** *	-0.311	0.152	1						
**Plasma adiponectin**	0.303	**0.409** *	-0.128	0.225	**0.733** **	1					
**Dams’ caloric intake**	**0.452** *	**0.478** **	0.067	0.009	0.047	-0.044	1				
**Dams’ weight (day 15)**	**0.531** **	**0.501** **	0.147	0.340	**0.365** *	**0.402** *	0.358	1			
**Dams’ weight increase**	-0.129	-0.341	0.057	**0.436** *	-0.096	-0.009	**-0.434** *	0.305	1		
**Male pups’ weight increase**	0.286	0.163	-0.078	0.071	0.036	0.055	**0.697** **	0.192	-0.245	1	
**Female pups’ weight increase**	0.136	0.020	-0.034	0.078	-0.075	-0.033	**0.593** **	0.062	-0.169	**0.860** **	1

*Significant correlation at p<0.05 level,

**significant correlation at p<0.01 levels (bilateral).

**Fig 5:**
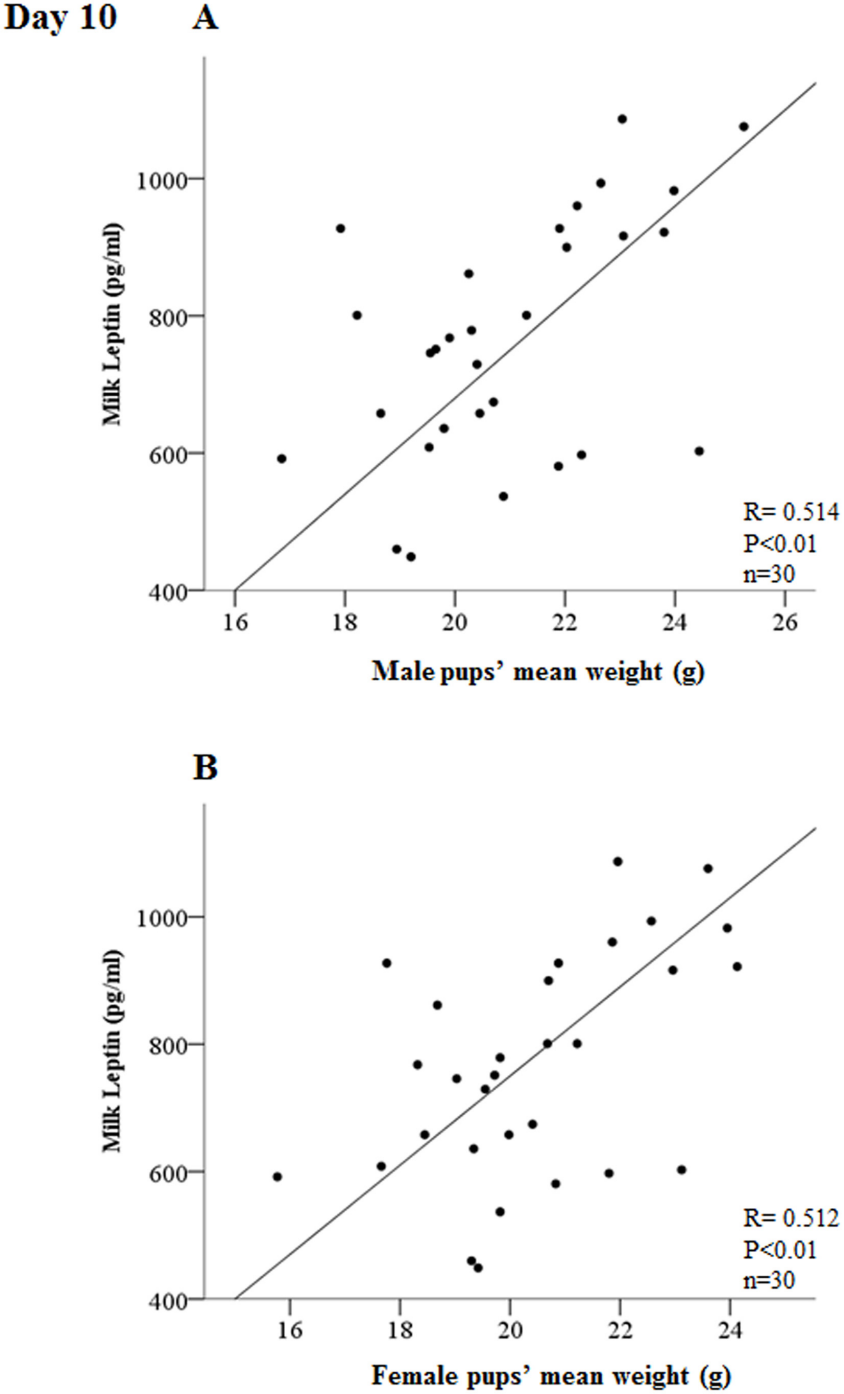
Correlation between milk levels of leptin with the mean weight of male (A) and female (B) pups at day 10 of lactation. The value of the Pearson’s correlation index (R) and level of significance (p) is given in the figure. The mean weight of the pups was calculated for males and females in each litter (n = 30).

Last, we also performed a correlation analysis of the levels of the three hormones in the milk (every different day studied) of each mother with the mean weight of the male and female pups of their litters. We only found a highly significant correlation (for both male and female) at day 10 between milk leptin levels and pup´s mean weight (p<0.01) (Fig 5) and a less significant correlation (p<0.05) between adiponectin levels and female-pup´s mean weight, also at day 10 (data not shown).

## Discussion

The first question formulated in this work was the possible existence of daily rhythms in the levels of leptin and other two important hormones present in mammalian milk (ghrelin and adiponectin) and the answer is that we have found rhythms in the three hormones in milk, but not daily, since they have a period of about half a day, and only at the earliest phase of lactation (day 5 in our study) (Figs 1–3). This period of about half a day (12h) suggests that these rhythms at day 5 fit with a circasemidian pattern, and the possibility that they may be controlled by circadian clocks arises, as reported by other authors for other molecules and tissues [41], an interesting point that would deserve more research. Moreover, the highest positive correlation between milk and dam´s plasma levels of leptin and adiponectin is shown at day 5 (Table 1), and even for ghrelin day 5 is the only day where there is a correlation (also positive). Although both leptin and adiponectin milk levels are still positively correlated with their plasma levels in dams at days 10 and 15 (Tables 2 and 3), this level of correlation is lower; moreover, at day five the shape of the curve for the three hormones in milk is someway similar to the shape of the curve representing the plasma levels (Figs 1A, 2A and 3A). Hence, the results suggest that at the earliest phase of lactation is when the milk levels of leptin, ghrelin and adiponectin can be more dependent on their circulating levels in the dams, but only in part, since we observe significant rhythmicity only in the milk levels which is nor present in the circulating levels. Moreover, this can be put into context for each hormone as following.

In the case of leptin, at day 10 not daily changes in milk levels were observed at all, showing very constant levels throughout the day, in contrast to the plasma levels in dams, which showed a significant increase during the day, specially towards the beginning and during the first hours of the dark phase (Fig 1B) (although the changes were not enough to observe a significant daily rhythm); a similar result for plasma levels was shown at day 15, but in this case the changes show a true daily rhythm in circulating leptin (Fig 1C). Thus, considering leptin´s anorexigenic function, the circadian fluctuations of plasma leptin may be related with food intake, as reported in a previous study with male rats [42]. On the contrary, at day 5 we observe a partial stability in plasma leptin levels during the light period, decreasing at 24:00, which is a different pattern, thus suggesting that lactating dams at day 5 may show this difference due to their environmental condition (early phase of lactation). According to this, Pickavance et al. [43] described that the normal diurnal rhythm shown in non-lactating (and non-pregnant) control rats in serum leptin (with higher levels in the dark phase) is lost in lactating rats (which do not show differences between light and dark phase), so this fits with our results; nevertheless, they used a group of lactating females between days 12–15 of lactation for their measurements and in our experiment we already observe a normalization of the cycle at day 15 of lactation. At any case, the results suggest that dams would change to a pattern more similar to non-lactating females in the cycle of circulating leptin levels when the lactation is advanced and the pups have significantly grown (and they start to eat chow diet before the end of lactation [44, 45]). On the other hand, despite the positive correlation between milk and plasma leptin levels at days 10 and 15, the rhythm analysis suggests that at the intermedium and advanced points of lactation the regulation of milk leptin concentration may be quite independent of the circulating levels and it may be related with the evolution or maturation of the mammary gland during lactation.

Curiously, when we compare the mean levels of leptin concentration in milk and plasma at the different days studied (Fig 4), there are no differences along lactation in the circulating levels in the dams, but there is a marked and significant increased peak of milk leptin concentration at day 10, coinciding with the day where no circadian changes were found in milk and where the milk and plasma leptin curves were quite different. In the literature, there are controversial results since an increase in milk leptin along lactation [9] or not significant changes in control lactating rats [46] (both measured at days 7, 14 and 21) have been described, although these studies did not follow a circadian design as the one used here. At this point, one important idea to highlight is the concept of leptin surge: Rayner and colleagues described in 1997 an increased peak in the circulating levels of the rat pups in the neonatal period [20], also shown in mice [19], and Ahima and colleagues in 1998 coined the term “leptin surge” as an increased peak of plasma leptin concentration in mouse pups during the neonatal period which happens between days 7–10 and which is thought to be crucial in postnatal development and function of the neuroendocrine axis [18]. Some authors have suggested that the leptin surge in mice pups could reflect the high fat content of diet (milk) during the suckling period [19], although this explanation has been discussed since no rise in circulating triglycerides or body fat in such period was reported in the model of Ahima and colleagues [18]; in this sense, our data suggest that leptin milk content may be important. To our knowledge, this is the first time that the increased peak of leptin is described in the milk of lactating mothers and this observation raises an interesting hypothesis: the leptin surge in the pups may be directly related with a leptin surge in the milk. Thus, not only the possible biological rhythms (specially at the beginning of lactation) in milk should be considered important, but also the increase in milk leptin around the mid of lactation may be relevant in the development and metabolic programming of the progeny. We must remember that oral leptin has been demonstrated to be absorbed undigested by the immature gastric epithelium of the rat neonates exerting biological effects [9, 39]. Other interesting datum of the present study which enhances the interest of this question is the fact that the only one of the three hormones studied here whose levels in milk are highly correlated with both male and female pup´s weight is leptin, and this correlation is only found at day 10 of lactation (Fig 5), further highlighting the importance of the milk leptin surge reported here.

With slight increases along lactation, the mean values of milk ghrelin levels have not shown significant differences among the three days studied (5, 10, 15), but show a slight tendency to decrease in plasma (Fig 4). As already explained above, we have not observed a daily rhythm in milk ghrelin at days 10 and 15, but there was rhythm at day 5 with a period of about half a day, with an increased peak at 16:00, reflecting a pattern similar to the plasma time-curve, and another at 4:00, together with a positive correlation with plasma levels only at day 5, but not at days 10 and 15. Thus, milk ghrelin levels seem to be poorly dependent on plasma levels at mid or late lactation and partially dependent at early lactation. As suggested for leptin, it could be proposed that ghrelin secretion in the mammary gland itself (since breast tissue expresses it and has been suggested to produce and secrete it [26, 47]) could be the main or an important source of ghrelin in the mid and late lactation period. Using the same reasoning used for leptin above, we can suggest that at day 10 and thereafter the mammary gland is more mature and can adapt to the pup´s requirements by regulating ghrelin secretion into the milk more independently of its changes in circulation. On the other hand, compared to leptin and ghrelin, the correlation of milk adiponectin with its plasma levels is higher (given by the Pearson’s correlation index in Tables 1–3 and by the similarity of the shapes of the curves in Fig 3) and, moreover, milk and circulating adiponectin levels are much higher than those of leptin and ghrelin (Fig 4), as expected [5] and they decrease along lactation (in accordance with results obtained in humans [32]). At any case, what is relevant in our study is the fact that at day 5 there is significant rhythmicity in milk adiponectin levels (again, with and bimodal pattern, like leptin and ghrelin at day 5), partly reflecting circulating changes (specially at day 5), while the levels become very constant later on, especially at day 15, both in milk and plasma. Considering the high concentration of adiponectin in milk and its suggested role in neonatal development and metabolic programming [5, 34], the significance and the consideration of the reported rhythms should be taken into account in future experiments or applications. With respect to the relationship between leptin and adiponectin, other authors have described an inverse correlation of their plasma levels in humans [48, 49], but a positive significant correlation between both leptin and adiponectin circulating levels in healthy infants [50], thus suggesting that the regulation and relation between these two hormones may be different in adults with respect to individuals during the perinatal period; our results, although obtained in the mothers (adult individuals), would be in the same line as the correlation found in infants, suggesting a possible different relationship between the levels of the two hormones depending on the physiological state (in this case, lactation). The results also show a general positive correlation of milk leptin and adiponectin concentrations (Tables 1–4), especially during the first half of lactation, suggesting a parallel regulation, at least partially, of their milk levels (but without surge for adiponectin levels).

The results also bring about other interesting relationships, such as the one of the levels of milk and plasma leptin and adiponectin with the weight attained by the dams (Table 4). In the case of plasma ghrelin, it is positively correlated with dam´s weight increase, a fact that would agree with the orexigenic role of ghrelin [51]. The multiple correlation analysis also shows a clear positive relationship between dam´s calorie intake and the growth (weight increase) of the pups (both male and female), which makes sense, while there is no correlation of the pup´s weight increase with the mean levels in milk throughout lactation of any of the hormones studied; nevertheless, we must remember, as discussed above, that at day 10 there is a highly significant positive correlation between milk leptin levels and the weight of the pups (male and female) (Fig 5) coinciding with the leptin surge observed in milk (Fig 4) and the plasma (pups) leptin surge described in the bibliography [18–20].

## Conclusions

In summary, we describe here the existence of rhythms in the hormone concentration, with a period around half a day, of milk leptin, and also ghrelin and adiponectin, only at the early phase of lactation, a new knowledge that is important and that may have a significant influence in the development of the progeny. The possibility that these rhythms may be related with circasemidian rhythms in the pups may not be ruled out and it may deserve more research. We also describe the existence of a “milk leptin surge” around mid-lactation (day 10) which may be important for the known leptin surge described in the circulation of pups and also crucial in the development of pups and metabolic programming. The biological rhythms of leptin and other milk hormones such as ghrelin and adiponectin should be taken into account in future research and applications, since it may influence the development of formula milks adapted to the phase of lactation (e.g. in early lactation) that could take into account the possibility of biological rhythms in key milk hormones.
